# Collected Thoughts on Mycobacterial Lipoarabinomannan, a Cell Envelope Lipoglycan

**DOI:** 10.3390/pathogens12111281

**Published:** 2023-10-26

**Authors:** Jordi B. Torrelles, Delphi Chatterjee

**Affiliations:** 1 International Center for the Advancement of Research and Education (I • Care), Texas Biomedical Research Institute, San Antonio, TX 78227, USA; 2 Population Health Program, Texas Biomedical Research Institute, San Antonio, TX 78227, USA; 3 Mycobacteria Research Laboratories, Department of Microbiology, Immunology and Pathology, Colorado State University, Fort Collins, CO 80523, USA

**Keywords:** tuberculosis, *Mycobacterium tuberculosis*, lipoarabinomannan, lipoglycan, mycobacteria

## Abstract

The presence of lipoarabinomannan (LAM) in the *Mycobacterium tuberculosis* (*Mtb*) cell envelope was first reported close to 100 years ago. Since then, numerous studies have been dedicated to the isolation, purification, structural definition, and elucidation of the biological properties of *Mtb* LAM. In this review, we present a brief historical perspective on the discovery of *Mtb* LAM and the herculean efforts devoted to structurally characterizing the molecule because of its unique structural and biological features. The significance of LAM remains high to this date, mainly due to its distinct immunological properties in conjunction with its role as a biomarker for diagnostic tests due to its identification in urine, and thus can serve as a point-of-care diagnostic test for tuberculosis (TB). In recent decades, LAM has been thoroughly studied and massive amounts of information on this intriguing molecule are now available. In this review, we give the readers a historical perspective and an update on the current knowledge of LAM with information on the inherent carbohydrate composition, which is unique due to the often puzzling sugar residues that are specifically found on LAM. We then guide the readers through the complex and myriad immunological outcomes, which are strictly dependent on LAM’s chemical structure. Furthermore, we present issues that remain unresolved and represent the immediate future of LAM research. Addressing the chemistry, functions, and roles of LAM will lead to innovative ways to manipulate the processes that involve this controversial and fascinating biomolecule.

## 1. Introduction

The hallmark of *Mycobacterium tuberculosis* (*Mtb*) is a complex hydrophobic lipid-rich cell envelope [1]. The dominant features have been studied by electron microscopy [2] and biochemical fractionation studies [3]. The *Mtb* cell envelope is mainly composed of four distinct entities [3]. Surrounding the cytosol is the cytoplasmic membrane or innermost membrane, which is similar to other bacterial membranes in thickness and mainly composed of proteins and phospholipids such as phosphatidic acid (PA), diphosphatidylglycerol (PG), phosphatidylcholine (PC), phosphatidylethanolamine (PE), and the phosphatidyl-*myo*-inositol mannosides (PIMs). The cell envelope lipoarabinomannan (LAM) and lipomannan (LM) are lipoglycans found anchored in the plasma membrane via a phosphatidyl-*myo*-inositol moiety that is the structural basis of the PIMs. Beyond the cell membrane, peptidoglycan (PG) and its attached arabinogalactan (AG), which together form the AGP complex (AGP), are apparently located in the periplasmic space. Outermost is an outer membrane or “*mycomembrane*”, which consists of the mycolic acids that form a distinct layer [4]. This peripheral lipid-rich cell envelope and its distinct components have been defined as virulent factors due to their ability to trigger a host response detrimental to the host and favoring the establishment of an *Mtb* infection. A debate still exists on the exact location of the PIMs, LM, and LAM. The latter may be anchored in the plasma membrane during its biosynthesis but also exposed on the cell envelope surface. Evidence of the existence of “*kettle holes*” on the cell envelope poles, which could allow the surface exposure of plasma membrane anchored LAM [5,6,7,8], could explain this seeming contradiction. The concept of the exposure of LAM on the surface is supported by quantitative transmission electron microscopy studies indicating that LAM is mainly located in the bacillus poles/tips and within cell envelope surface depressions [9,10,11]. Meanwhile, the concept of LAM anchored to the plasma membrane is supported by the need for mechanical and chemical steps to extract LAM from the bacterial cell envelope [12] and by the fact that many steps in the LAM biosynthesis are associated with the plasma membrane [13,14].

## 2. Defining the Basic Structure of LAM: A Brief History

**Figure 1:**
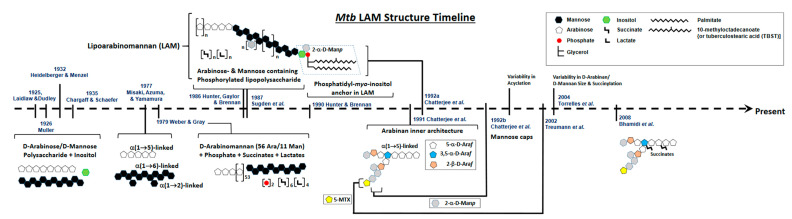
Schematic Representation of the evolution of *Mtb* LAM structural studies. Approximate timeline for last 100 years starting in 1925.

The volume of literature concerning the discovery of LAM, followed by its structural characterization and discovery of its immunological properties, is substantial. A timeline of the evolution of LAM structural studies is presented in Figure 1. Briefly, in 1925, Laidlaw and Dudley [15] used a mild alkaline extraction of a defatted human strain of *Mtb* to obtain a novel component that contained 31% pentose. Later, Heidelberger and Menzel using dilute acetic acid on defatted *Mtb* [16], as well as Laidlaw and Dudley and Mueller using culture filtrate, showed the presence of a serologically active polysaccharide rich in D-arabinose and D-mannose [17]. The presence of this polysaccharide was confirmed further in avian and bovine strains [18]. Over the next 10 years, several studies followed, all concluding the presence of a certain polysaccharide composed of D-mannose, D-arabinose, and an unidentified sugar acid that reacted as haptens.

The potential first evidence of a mycobacterial LAM-like structure was established in 1935 by Chargaff and Schaefer, who identified the polysaccharide, designated as fraction A, as being a soluble, dextrorotatory, weak acid containing 77% reducing sugars and 3% amino sugars, mainly composed of mannose and arabinose together with small amounts of inositol [19]. Since the time that this study was reported, many subsequent studies focused on defining the chemical and immunological properties of this polysaccharide, including its α-haemosensitin properties involved in the Middlebrook–Dubos hemagglutination reaction.

In 1977, a serologically active and LAM-related D-arabino-D-mannan from *Mtb* was characterized with a 2:1 ratio of arabinose:mannose. This arabinomannan consisted of α-(1 → 5)-linked D-arabinose residues and α-(1 → 6)- and (1 → 2)-linked D-mannose residues. Further methylation and enzymatic degradation studies using *Arthrobacter* sp. α-D-mannosidase and M-2 enzyme (D-arabinan hydrolase) provided evidence of the existence of short side chains built up from α-(1 → 2)-D-mannosidic linkages that were attached to an α-(1 → 6)-linked mannan backbone [20].

In 1979, Weber and Gray [21] isolated an acidic arabinomannan from *M. smegmatis* and defined the presence of 56 arabinosyl and 11 mannosyl residues, 2 phosphates, 6 mono-esterified succinates, and 4 ether-linked lactate groups. Subsequently, this acidic polysaccharide was separated into phosphorylated and non-phosphorylated forms with similar structures, wherein the main structural feature was the presence of chains of contiguous arabinofuranosyl residues linked α-(1 → 5).

Close to 50 years since the first reported evidence of a polysaccharide containing arabinose, mannose, inositol, and phosphorous in *Mtb*, in 1986, Hunter et al. structurally defined a family of major arabinose- and mannose-containing phosphorylated lipopolysaccharides isolated from *Mtb* and *M. leprae* [22]. LAM-B, as designated, eluting off an ion exchange column, in addition to arabinose and mannose, also contained glycerol and a *myo*-inositol 1-phosphate, as well as acylations with lactate, succinate, palmitate, and 10-methyloctadecanoate. Other studies followed that provided details of the arabinose-to-mannose ratios and the presence of palmitic, stearic, and tuberculostearic acids as the main fatty acids [23]. Brennan and colleagues, when characterizing the fully acetylated PIM family and their biosynthesis, realized that LAM was essentially a polyglycosylated extension of the PIMs [24,25]. Confirmation was provided in 1990 by Hunter and Brennan, who established the presence of the phosphatidyl-*myo*-inositol membrane anchor present in the PIMs in *Mtb* LAM and LM [26]. The most decisive feature of this study was the evidence for the presence of glycosidically linked diacylglycerol residues, in common with the phosphatidylinositol (PI) of the PIMs [24].

**Figure 2:**
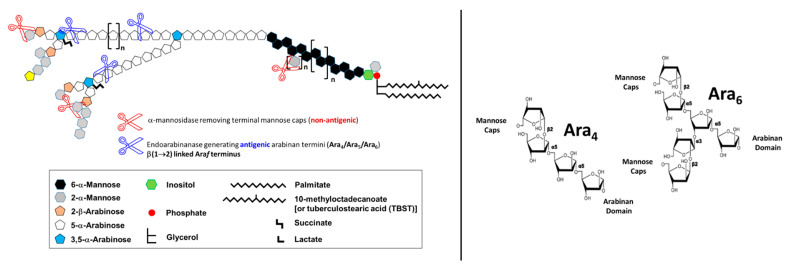
Schematic representation of ManLAM from *Mtb*. The antiLAM monoclonal antibodies (mAbs) CS-35, A194-01, and CHCS9-08 are shown to react with the LAM arabinan terminal arrangements (Ara_4_ and Ara_6_, respectively). These three mAbs are widely used in immunoassays for TB diagnosis. The cartoon is based on a screening of 12 synthetic arabinan glycoconjugates by Dr. Todd Lowary [40]. In order to identify which motifs of LAM are being recognized by anti-LAM antibodies, an exhaustive digestion of LAM with commercially available α-mannosidase (derived from Jack Beans) is performed. This digestion removes the mannose caps of LAM (depicted as grey hexagons). An additional digestion using an endo-arabinanase (in-house isolated from *Cellulomonas*) releases arabinan fragments from the D-arabinan domain, mainly Ara_4_, Ara_5_, and Ara_6_ fragments. These enzymatic digestions allowed the identification of the Ara_4_ and Ara_6_ motifs as the ones being recognized by monoclonal antibodies against LAM and were used in the development of TB diagnosis downstream.

**Figure 3:**
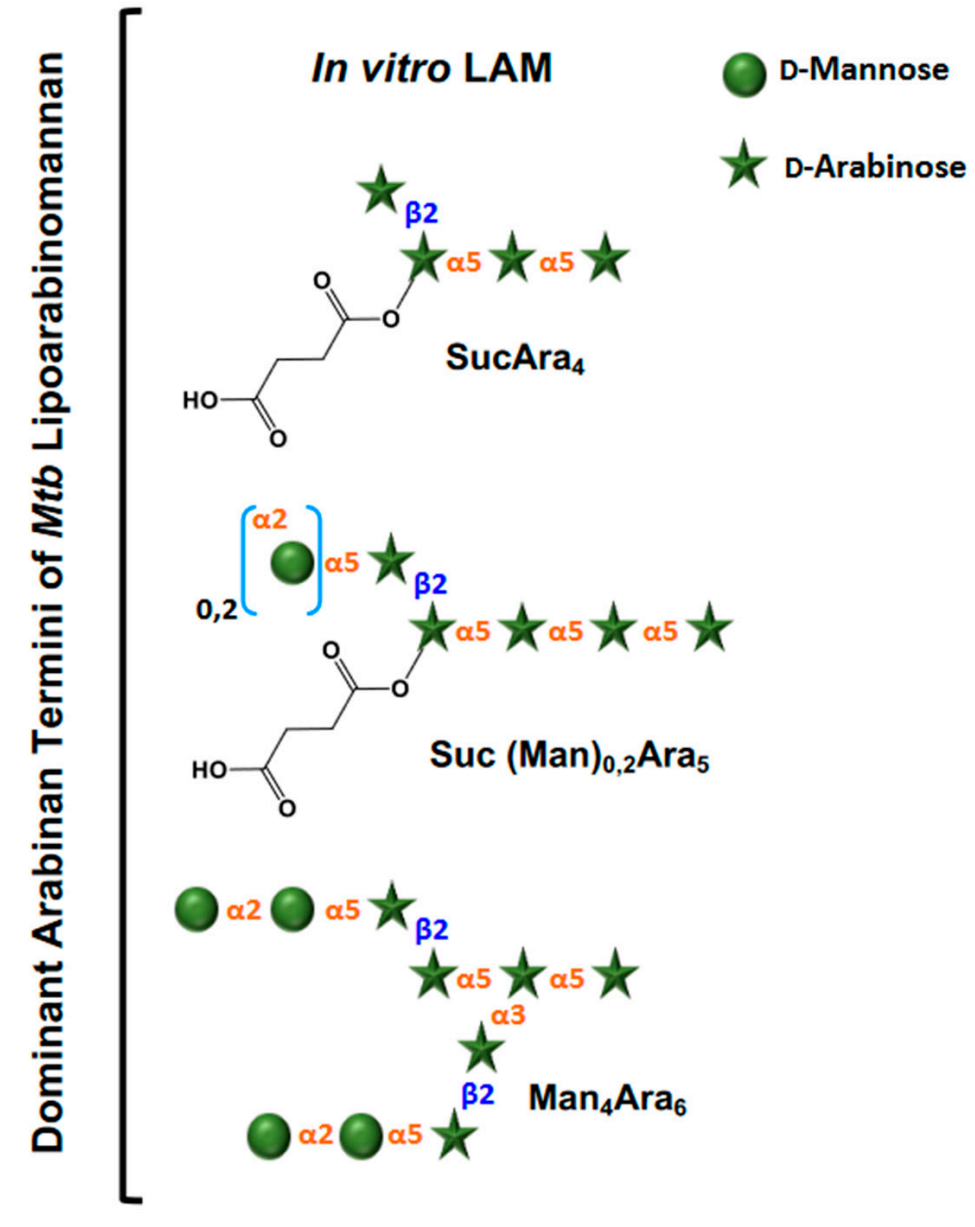
Dominant arabinan termini of *Mtb* lipoarabinomannan from culture. Only non-reducing ends are shown. The terminal structures evolved after extensive enzymatic degradation of LAM followed by liquid chromatography with tandem mass spectrometry (LC/MS-MS) analyses [41]. Abbreviations: Ara = D-Arabiniose, Man = D-Mannose, Suc = Succinates.

Chatterjee and colleagues, using enzymatic treatments, subsequently focused on the exact architecture of the arabinan domain, establishing that its internal regions consist of branched 3,5-linked α-D-Ara*f* units with stretches of linear 5-linked α-D-Ara*f* residues attached at both branch positions, whereas the non-reducing terminal segments of the LAM arabinan domain consist of either of the two arrangements, Ara_4_: β-D-Ara*f*-(1 → 2)-α-D-Ara*f*-(1 → 5)-α-D-Ara*f*-(1 → 5)-α-D-Ara*f* → or Ara_6_: [β-D-Ara*f*-(1 → 2)-α-D-Ara*f*-(1 → ]_2_ → (3 and 5)-α-D-Ara*f*-(1 → 5)-α-D-Ara*f* → [27] (Figure 2). Two subsequent studies by Chatterjee and colleagues further refined the LAM structure. The first focused on describing the structural environment of the PI anchor, showing clearly the presence of the 1-(sn-glycerol-3-phospho)-D-*myo*-inositol-2,6-bis-α-D-mannoside unit, indistinguishable from that derived from phosphatidyl-*myo*-inositol dimannoside (PIM_2_). The same study also demonstrated that the C-6 position of inositol is the site of attachment of the mannan core of LAM, which consists of an α(1 → 6)-linked backbone with several α(1 → 2)-side chains [28]. In a parallel study, Chatterjee and colleagues also demonstrated that the termini of LAM from virulent *Mtb* strains, unlike those from attenuated *Mtb* strains, are extensively capped with mannosyl (Man*p*) residues, either with a single α-D-Man*p*, a dimannoside (α-D-Man*p*-(1 → 2)-α-D-Man*p*) or a trimannoside (α-D-Man*p*-(1 → 2)-α-D-Man*p*-(1 → 2)-α-D-Man*p*). Thereby, the functionally important so-called mannose-capped LAM or Man-LAM was identified (Figure 3). Indeed, after this work, a large number of studies followed, uncovering a wide array of biological properties attributable to the various forms of *Mtb* LAM has, and how *Mtb* LAM compares to LAMs from other *Mtb* complexes and mycobacterial species [29,30,31,32,33]. In particular, these subsequent studies, using advanced HPLC resolution and MS analysis, demonstrated the enormous intrinsic heterogeneity and complexity of LAM at both the reducing and non-reducing ends, as well as variations in the fatty acyl content of the GPI anchor. Variations in the length and branching patterns of the D-mannan and D-arabinan domains were also observed. These, and potentially other short-chain acyl features, have now been established as attached to the arabinan domain. Further, diversity within the Man-caps, in particular, the presence of a 5-deoxy-5-methylthio-xylofuranose (MTX) residue, has been observed [34,35,36]. Finally, studies also depicted the effects of specific drugs (e.g., ethambutol) on the structure of LAM in drug-resistant *Mtb* strains and other mycobacterial species, as well as how the size of LAM influences *Mtb*/host cell interactions [30,37,38]. Current efforts are directed towards advancing the native structure of LAM isolated from tissues and/or specimens, as well as developing new tools for the structural characterization of LAM [34,39].

## 3. Evolution of LAM Structural Studies across Species

Our knowledge of the structure of LAM is constantly evolving [41,42], and although there are differences across species, the overall features are common to all, at least in all those investigated to date. Essentially, LAM throughout contains four structural domains: a mannosylated phosphatidyl *myo*-inositol (PI) anchor, a D-mannan core, a D-arabinan domain, and different capping motifs that contribute to species and strain diversity (reviewed in [43,44]). The mannan core consists of a chain of α-(1 → 6)-linked mannopyranose (Man*p*) residues, some of which are modified by the addition of α-(1 → 2)-linked Man*p* motifs, usually, but not always, as a single residue. An arabinan, composed of solely D-arabinofuranose (Ara*f*) residues, is attached to the non-reducing end of the mannan core [42]. Capping motifs can be added at specific positions contributing to intra- and inter-species structural variability [41]. Fast-growing mycobacterial species predominately produce AraLAM (uncapped LAM) or PILAM (phosphoinositol capped, as defined for *M. smegmatis*) [45]. Slow-growing mycobacteria like *Mtb* and *M. leprae* produce LAM with α-(1 → 2)-linked Man*p* capping residues, giving a molecule referred to as ManLAM [8,30]. Within the *Mtb* complex group, variations regarding primarily the degree of terminal mannose capping present in ManLAM can range between 40–70% [27,28,46,47,48]. Some fast-growing and/or non-pathogenic mycobacteria also produce ManLAM; however, these differ in the Man content of the capping motifs. In addition to Man*p* capping, ManLAM from strains of the *Mtb* complex group also contains a unique residue –MTX on the terminal Man*p* caps [34,35,36,49]. Further variability comes from acylation of the arabinan, most commonly with succinylation (Figure 3), which can be found either in the internal [50] or termini [41] of the arabinan domain [51].

**Figure 4:**
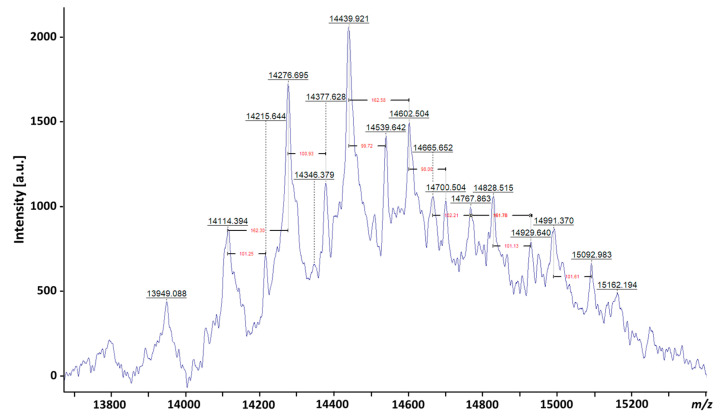
MALDI-TOF analysis of LAM from *Mtb*. The mass spectrometry (MS) was done on Bruker ultrafleXtreme matrix-assisted laser desorption time-of-flight spectrometry (MALDI-TOF/TOF MS), indicating a molecular mass for ManLAM of approximately 15 kDa. Heterogeneity in mass of 162 *m*/*z* corresponds to a hexose and 100 *m*/*z* corresponds to succinates.

The extreme heterogeneity in LAM is evident from the broad diffuse band observed on a SDS-polyacrylamide gel electrophoresis (PAGE) analysis of LAM and LM [22] and its capacity to be separated in different isoforms [31], as well as from several recent matrix-assisted laser desorption/ionization—mass spectrometry (MALDI-MS) studies, which provided an indication of the mean distribution of true molecular mass. It has been shown that native LAM from *M. bovis* BCG and *Mtb* gives a broad peak centered at 17.3 kDa before deacylation and 16.7 kDa after deacylation, with a reported size distribution range of ±4 kDa depending on the studied strains [52]. With recent advances in MS instrumentation, a peak centered at *m*/*z* 14439.921, providing a molecular mass of approx. 15 kDa for LAM, has been reported (Chatterjee et al., personal communication) (Figure 4).

## 4. Biological Properties of *Mtb* LAM

*Mtb* LAM was widely studied in the 1990s. Studies started to reveal the immunological properties of LAM, initially as a potential candidate for ELISA-based diagnosis of TB and leprosy [53,54], as well as its capacity to inhibit the activation of macrophages, among other immunological features [55,56].

Early data published include LAM-induced abrogation of T-cell activation [57]; inhibition of various IFN-γ-induced functions including macrophage microbicidal and tumoricidal activity [56]; scavenging of potentially cytotoxic oxygen free radicals [58]; inhibition of protein kinase C activity [58]; and evocation of a large array of cytokines associated with macrophages such as TNF [59,60,61,62,63], GM-CSF, IL-1α, IL-1β, IL-6, and IL-10 [64,65]. In 1991, Chan and colleagues provided the first evidence of the role of *Mtb* LAM in downregulating macrophage effector functions by scavenging potentially cytotoxic oxygen free radicals, inhibiting protein kinase C activity, and blocking the transcriptional activation of IFN-γ inducible genes [58]. Related to the use of LAM by *Mtb* to recognize and infect host cells, it has been reported that both the mannose receptor (MR) and DC-SIGN in phagocytes recognize and bind to LAM, and this binding can result in efficient internalization of *Mtb* to its intracellular niche within host cells [9,66].

Of the many biological properties that *Mtb* ManLAM has exhibited, one has led us to understand how critical this molecule is for the persistence of *Mtb* within host cells. First, Schlesinger and colleagues showed that ManLAM is recognized by macrophages [67]. This finding was later supported by others [68]. Subsequently, Deretic and others showed the capacity of *Mtb* ManLAM to block phagosome acidification [69], and later, Schlesinger and colleagues showed how *Mtb* exploits the ManLAM/MR route [70] to gain entry into macrophages and survive [9], potentially generating the perfect niche for survival and subsequent immune responses deriving into granuloma formation and thus, contributing to the persistence of *Mtb* in a latent stage in the host. Another well-known characteristic of ManLAM is its intracellular processing and presentation towards CD1-restricted T cells, contributing to the host immune response against *Mtb* infection [31,71,72]. In this regard, liposomal delivery of ManLAM triggers ManLAM-specific T cells [73]. The ManLAM phosphatidyl-*myo*-inositol moiety plays a central role in its binding to CD1b, although the exact epitopes involved in this binding have not yet been defined [31]. Thus, ManLAM is shown to be a CD1b ligand and some of its in vivo properties are probably derived from the binding of CD1-restricted T cells [71]. Further details on LAM biological functions are described in detail elsewhere [43].

## 5. LAM as a Diagnosis Biomarker for TB Disease

*Mtb* LAM represents up to 15% of the bacterial mass [1,26,74,75]. This molecule is firmly but non-covalently attached to the inner membrane and extends to the exterior of the cell envelope [76], where it interacts as a potent virulence factor modulating host immune responses [77]. Importantly, the linear terminus Ara_4:_ (β-D-Ara*f*-(1 → 2)-α-D-Ara*f*-(1→5)-α-D-Ara*f*-(1 → 5)-α-D-Ara*f*) (Figure 2) and a branched terminus Ara_6_: ([β-D-Ara*f*-(1→2)-α-D-Ara*f*-(1-)_2_ → 3, and →5]-α-D-Ara*f*-(1→5)-α-D-Ara*f*) (Figure 2) are shown to be the epitopes recognized by anti-LAM monoclonal antibodies (mAb) [40,74,78,79,80,81].

LAM has been validated to be present in variable concentrations in sputum, serum, and urine [82,83,84]. In recent years, several laboratories including ours have made significant advances toward developing urinary LAM-based diagnostics for active TB [40,79,85,86,87]. A point-of-care (POC) test that readily diagnoses active TB would reduce diagnostic delays, interrupt transmission with appropriate therapy, and address many of the current gaps in global TB control (Stop TB Partnership in collaboration with Imperial College London). The development of sensitive POC methods to detect LAM in non-invasive samples such as urine using immunoassays is currently stagnant due to the suboptimal sensitivity of the assays. Current methods are also limited in applicability to TB diagnosis only in people living with HIV and severe disease [84,88,89]. However, there are studies showing that LAM can be also detected in urine from active TB cases without HIV co-infection [85,90]. Recently, as an alternative to urine, groups have focused their attention on other non-invasive samples such as exhaled breath condensate (EBC), in which ManLAM seems to be present in significant amounts [91,92].

We and the others actively pursuing the field have hypothesized that these disappointing results with POC methods are due to the array of anti-LAM antibodies used in the current commercially available POC immunoassays, which are generally raised against one common *Mtb* laboratory strain [40,79,87,93]. Thus, *Mtb* lineage-specific and demographical strain anti-LAM antibodies may be required to develop more efficient LAM detection-based POC diagnostic tests for their use in TB-endemic countries.

We should also add that the structure of LAM differs among mycobacterial species and strains. In the context of non-typical mycobacteria (NTM) such as *M. avium*, from the few studies performed, the LAM structure differs mainly in the degree of mannose caps present. Although *Mtb* presents tri-, di-, and mono-mannose-capped LAM, *M. avium* LAM mainly contains mono-mannose-capped LAM [48,49]. From the diagnosis perspective, LAM is well-established as a biomarker for active TB, with high sensitivity and specificity by immunological assays. Recently, urinary LAM was considered as a biomarker for determining cystic fibrosis (CF) patients at low risk of NTM infection [94]. In this regard, the urine LAM test is reported to have high specificity (91–99%) but low sensitivity (9–39%) for pulmonary NTM in the Danish CF population [95]. We have reported that CF patients with recent positive NTM sputum cultures have detectable amounts of LAM in their urine (measured by Gas Chromatography–Mass Spectrometry), with a 100% concordance between a non-detectable quantity of urine LAM and a history of negative NTM sputum culture. We have also reported urinary LAM being a sensitive marker of treatment response in an individual with *M. abscessus* successfully treated with phage therapy [96]. However, we should emphasize that in the NTMs and *M. abscessus* cases studied, immunoassays using TB-LAM-specific antibodies failed to detect LAM in urine, even after several-fold concentrations. We have speculated that there could be several reasons for this—that LAM is present albeit in very low amounts, or there are fundamental differences between TB-LAM vs. NTM-LAM structures, or how these are presented. While detecting low LAM concentrations in diluted urine is part of the challenge, a key gap in our existing knowledge remains related to which LAM recognition motifs are most critical for highly sensitive LAM detection.

As of today, LAM from NTM has not been studied in depth. Thus, it is not apparent if any of the features such as Man caps, MTX, or acylation are present/absent in NTM-LAM. Using well-phenotyped collected CF samples [94], we have tested anti-LAM monoclonal antibodies using CF urine samples with LAM concentrations previously determined by GC-MS. Dot blot screening indicated that antibodies have only moderate activity towards NTM-LAM compared to TB-LAM, despite a fairly high concentration (~250 ng/mL) of LAM spiked in the urine samples. This suggests that either the epitopes of NTM-LAM vs. TB-LAM are different, or unlike in TB, during NTM infection LAM is cryptic and is somehow not available to the antibodies for recognition due to biofilm formation. In either case, we will need to generate specific monoclonal and polyclonal antibodies to be able to increase the sensitivity of any immunoassay in detecting NTM-LAM [81].

## 6. Concluding Remarks

The structure and biological functions of LAM are still widely studied to understand its importance in the context of *Mtb* pathogenesis and drug resistance. Over time, new protocols have been developed to extract, purify, and accurately characterize the LAM molecule. Current knowledge on the structure of LAM has resulted primarily from detailed studies on a few selected laboratory strains of *Mtb* [31,74], *M. bovis* bacillus Calmette–Guérin (BCG) [29], *M. smegmatis* [32], and *M. kansasii* [97], among a few others. Efforts have been invested in correlating unique structural features with aspects of the immunopathogenesis of TB [74]. An outcome of these efforts is the consensus that the mannose caps of LAM (ManLAM) constitute the single most important structural entity engaged in phagocytosis by phagocytes and subsequent events such as inhibition of phagosome/lysosome fusion and immunomodulation of host responses.

Indeed, the presence of LAM on the cell surface allows *Mtb* to mimic mammalian glycoproteins that are cleared subsequently from circulation by phagocytes using specific receptors such as the MR through the recognition of the LAM mannose caps. This phenomenon confers on *Mtb* a unique pathway of entry and survival within host cells. However, how LAM is being processed metabolically within host cells and its role in *Mtb* intracellular survival over a long period is, as yet, not well defined. In this context, it is speculated that the ratio of mannose-containing molecules (PIMs, LM, ManLAM, AM, and mannosylated glycoproteins) on the *Mtb* cell envelope may play a role in mitigating host immune responses, as well as contributing to the bacillus dormancy metabolic status within host cells [98]. However, how the degree of surface mannosylation on different *Mtb* strains correlates to their pathogenesis and their development of drug resistance is still unknown.

Although the overall structure and properties of LAM are conserved, its chemical composition varies among *Mtb* complex species and *Mtb* strains. In this regard, the human-adapted *Mtb* complex exhibits a strong phylogeographical population structure, with some *Mtb* lineages occurring globally and others showing a strong geographical restriction [99,100]. Among these, lineages L2 and L4 are widely distributed in the world, with L2 dominating in East Asia. L1 and L3 are found mainly in regions around the Indian Ocean, and L5 and L6 seem restricted to West Africa, whereas L7 seems to be exclusively found in Ethiopia. We posit that *Mtb* strains from different lineages will have a wide spectrum of virulence, differently modulating host immune responses, and thus, determining *Mtb* bacillary load in patients with pulmonary TB. In this context, it will be important to perform LAM phenotype mapping in *Mtb* strains that cause TB outbreaks in TB-endemic geographical areas, and further examine whether any epidemiologically relevant LAM structural characteristics are associated with these strains [41].

We end this review with some additional thoughts. We have introduced a historical perspective on how several laboratories including ours, starting with problems in the field of structural definition, have gradually become involved in the issues of biosynthesis, immunology, pathogenesis, and finally, in TB diagnosis. One of the vital contributions that we bring to such progress is the sensitivity towards precisely defined structural LAM attributes. Much has been learned about the chemistry and biology of LAM and yet, this knowledge is still evolving, as it is obvious from this review. As an example, unlike many other known glycans, LAM arabinan is a homopolymer with no repeating units, and a single donor decaprenylphosphoryl arabinose is identified to be donating all of the arabinofuranose residues [101]. Its location and distribution within the *Mtb* cell envelope remain unresolved.

Studies indicate that LAM is found in urine, serum, EBC, and lung biopsies from TB patients [34,91,92,93,102]. The fact that LAM is found in the serum of TB patients means that it probably affects a wide variety of host cell populations, consequently influencing both innate and adaptive immune responses during *Mtb* infection, dormancy, subclinical TB, and progression to active TB disease. Future studies on delineating the roles of LAM in immunopathogenesis need to be focused on in vivo studies whenever possible, with the hope that we will eventually delineate the LAM structural motifs that contribute as virulence factors and/or protective epitopes in vivo.

We believe that, due to their chemistry, LAM molecules are key glycans sitting at the crossroads of many critical biological processes. As such, we (the authors) strongly deem that the very final goal of LAM research should be to have an “atomic architecture” of the LAM structure in which its relevant features are finely described with their biological functions dissected. In the coming years, accomplishing this complex but fundamental task will have a significant impact on advancing the TB field.

LAM biosynthesis (a topic not addressed in this review) is not trivial and encompasses a complex network of enzymes with high energy demand. Thus, the necessity for *Mtb* to produce LAM to survive seems apparent. To this day, there are many biological properties described for LAM, but it is still unclear if the presence of LAM in the *Mtb* cell envelope ultimately benefits the bacterium or the host. In this regard, *Mtb* strains with a highly mannosylated cell envelope, including LAM, are thought to be adapted to the host, driving the dormant stage of the infection. Thus, a question that remains is: are the innate and adaptive immune responses against LAM protecting the bacterium, allowing *Mtb* infection to perpetuate? In this context, we have shown that upon minimal contact (15 min) with human alveolar lining fluid, the *Mtb* cell envelope is modified by alveolar homeostatic hydrolytic activities releasing cell envelope fragments into the milieu [103]. As a result, *Mtb* loses up to 65% of its LAM exposed on the envelope surface [103]. This released LAM could be binding with alveolar soluble innate components such as surfactant protein D (SP-D) and mannose-binding lectin (MBL), blocking innate responses that ultimately favor *Mtb* survival. This is yet to be proven.

Little is known about the role of LAM in the early events of *Mtb* infection driving the formation of the granuloma. As a future perspective in LAM research, there is a need for improved methods for studying the LAM structure in vivo. As an unquestionable hot topic, innovative, although certainly challenging, procedures, able to deliver the relevant information, would provide a realistic picture of the actual structure of LAM without any chemical alterations possibly related to the current isolation methods used. This would be a giant leap forward in elucidating any links between LAM’s specific structural characteristics, its real biological functions, and the disease arising from its detection by the host immune system.

## Data Availability

Not applicable.

## References

[1] Brennan P.J., Nikaido H. (1995). The envelope of mycobacteria. Annu. Rev. Biochem..

[2] Dahl J.L. (2004). Electron microscopy analysis of Mycobacterium tuberculosis cell division. FEMS Microbiol. Lett..

[3] Chiaradia L., Lefebvre C., Parra J., Marcoux J., Burlet-Schiltz O., Etienne G., Tropis M., Daffé M. (2017). Dissecting the mycobacterial cell envelope and defining the composition of the native mycomembrane. Sci. Rep..

[4] Daffé M., Marrakchi H. (2019). Unraveling the Structure of the Mycobacterial Envelope. Microbiol. Spectr..

[5] Dulberger C.L., Rubin E.J., Boutte C.C. (2020). The mycobacterial cell envelope—A moving target. Nat. Rev. Microbiol..

[6] Gaylord H., Brennan P.J. (1987). Leprosy and the leprosy bacillus: Recent developments in characterization of antigens and immunology of the disease. Annu. Rev. Microbiol..

[7] McNeil M.R., Brennan P.J. (1991). Structure, function and biogenesis of the cell envelope of mycobacteria in relation to bacterial physiology, pathogenesis and drug resistance; some thoughts and possibilities arising from recent structural information. Res. Microbiol..

[8] Chatterjee D., Khoo K.-H. (1998). Mycobacterial lipoarabinomannan: An extraordinary lipoheteroglycan with profound physiological effects. Glycobiology.

[9] Kang P.B., Azad A.K., Torrelles J.B., Kaufman T.M., Beharka A., Tibesar E., DesJardin L.E., Schlesinger L.S. (2005). The human macrophage mannose receptor directs Mycobacterium tuberculosis lipoarabinomannan-mediated phagosome biogenesis. J. Exp. Med..

[10] Hett E.C., Rubin E.J. (2008). Bacterial growth and cell division: A mycobacterial perspective. Microbiol. Mol. Biol. Rev..

[11] Torrelles J.B., Cardona P.J. (2012). Broadening our view about the role of Mycobacterium tuberculosis cell envelope components during infection: A battle for survival. Understanding Tuberculosis—Analyzing the Origin of Mycobacterium tuberculosis Pathogenicity.

[12] Shi L., Torrelles J.B., Chatterjee D., Parish T., Brown A.C. (2008). Lipoglycans of Mycobacterium tuberculosis: Isolation, purification, and characterization. Mycobacteria Protocols.

[13] Besra G.S., Morehouse C.B., Rittner C.M., Waechter C.J., Brennan P.J. (1997). Biosynthesis of mycobacterial lipoarabinomannan. J. Biol. Chem..

[14] Kaur D., Guerin M.E., Skovierova H., Brennan P.J., Jackson M. (2009). Chapter 2: Biogenesis of the cell wall and other glycoconjugates of Mycobacterium tuberculosis. Adv. Appl. Microbiol..

[15] Laidlaw P.P., Dudley H.W. (1925). A specific pre-cipitating substance from tubercle bacilli. Br. J. Exp. Pathol..

[16] Heidelberger M., Menzel A.E. (1932). Specific and non-specific cell polysaccharides of the humantype of tubercle bacillus, H37. Proc. Soc. Exp. Biol. Med..

[17] Mueller J.H. (1926). A chemical study of the specific elements of tuberculin: II. The preparation of resi¬due antigen from old tuberculin. J. Exp. Med..

[18] Menzel A.E., Heidelberger M. (1939). Specific and non-specific cell polysaccharides of an avian strain of tubercle bacillus. J. Biol. Chem..

[19] Chargaff E., Schaefer W. (1935). A specific polysaccharide from the Bacillus Calmette-Guerin (BCG). J. Biol. Chem..

[20] Misaki A., Azuma I., Yamamura Y. (1977). Structural and immunochemical studies on D-arabino-D-mannans and D-mannans of Mycobacterium tuberculosis and other Mycobacterium species. J. Biochem..

[21] Weber P.L., Gray G.R. (1979). Structural and immunochemical characterization of the acidic arabinomannan of *Mycobacterium smegmatis*. Carbohydr. Res..

[22] Hunter S.W., Gaylord H., Brennan P.J. (1986). Structure and antigenicity of the phosphorylated lipopolysaccharide antigens from the leprosy and tubercle bacilli. J. Biol. Chem..

[23] Sugden E.A., Samagh B.S., Bundle D.R., Duncan J.R. (1987). Lipoarbinomannan and Lipid-free arabinomannan antigens of *Mycobacterium paratuberculosis*. Infect. Immun..

[24] Brennan P., Ballou C.E. (1968). Biosynthesis of mannophosphoinositides by *Mycobacterium phlei*: Enzymatic acylation of the dimannophosphoinositides. J. Biol. Chem..

[25] Brennan P.J., Ballou C.E. (1967). Biosynthesis of Mannophosphoinositides by *Mycobacterium phlei*: The family of dimannosylphosphoinositides. J. Biol. Chem..

[26] Hunter S.W., Brennan P.J. (1990). Evidence for the presence of a phosphatidylinositol anchor on the lipoarabinomannan and lipomannan of *Mycobacterium tuberculosis*. J. Biol. Chem..

[27] Chatterjee D., Bozic C.M., McNeil M., Brennan P.J. (1991). Structural features of the arabinan component of the lipoarabinomannan of *Mycobacterium tuberculosis*. J. Biol. Chem..

[28] Chatterjee D., Hunter S.W., McNeil M., Brennan P.J. (1992). Lipoarabinomannan. Multiglycosylated form of the mycobacterial mannosylphophatidylinositols. J. Biol. Chem..

[29] Prinzis S., Chatterjee D., Brennan P.J. (1993). Structure and antigenicity of lipoarabinomannan from *Mycobacterium bovis* BCG. J. Gen. Microbiol..

[30] Torrelles J.B., Khoo K.H., Sieling P.A., Modlin R.L., Zhang N., Marques A.M., Treumann A., Rithner C.D., Brennan P.J., Chatterjee D. (2004). Truncated Structural Variants of Lipoarabinomannan in *Mycobacterium leprae* and an Ethambutol-resistant Strain of *Mycobacterium tuberculosis*. J. Biol. Chem..

[31] Torrelles J.B., Sieling P.A., Zhang N., Keen M.A., McNeil M.R., Belisle J.T., Modlin R.L., Brennan P.J., Chatterjee D. (2012). Isolation of a distinct *Mycobacterium tuberculosis* mannose-capped lipoarabinomannan isoform responsible for recognition by CD1b-restricted T cells. Glycobiology.

[32] Khoo K.-H., Dell A., Morris H.R., Brennan P.J., Chatterjee D. (1995). Inositol phosphate capping of the nonreducing termini of lipoarabinomannan from rapidly growing strains of *Mycobacterium*. J. Biol. Chem..

[33] Petzold C.J., Stanton L.H., Leary J.A. (2005). Structural characterization of Lipoarabinomannans from Mycobacterium tuberculosis and Mycobacterium smegmatis by ESI mass spectrometry. J. Am. Soc. Mass Spectrom..

[34] De P., Shi L., Boot C., Ordway D., McNeil M., Chatterjee D. (2020). Comparative Structural Study of Terminal Ends of Lipoarabinomannan from Mice Infected Lung Tissues and Urine of a Tuberculosis Positive Patient. ACS Infect. Dis..

[35] Treumann A., Xidong F., McDonnell L., Derrick P.J., Ashcroft A.E., Chatterjee D., Homans S.W. (2002). 5-Methylthiopentose: A new substituent on lipoarabinomannan in *Mycobacterium tuberculosis*. J. Mol. Biol..

[36] Turnbull W.B., Shimizu K.H., Chatterjee D., Homans S.W., Treumann A. (2004). Identification of the 5-methylthiopentosyl substituent in *Mycobacterium tuberculosis* lipoarabinomannan. Angew. Chem. Int. Ed. Engl..

[37] Torrelles J.B., Knaup R., Kolareth A., Slepushkina T., Kaufman T.M., Kang P.B., Hill P., Brennan P.J., Chatterjee D., Belisle J.T. (2008). Identification of mycobacterium tuberculosis clinical isolates with altered phagocytosis by human macrophages due to a truncated lipoarabinomannan. J. Biol. Chem..

[38] Zhang N., Torrelles J.B., McNeil M.R., Escuyer V.E., Khoo K.H., Brennan P.J., Chatterjee D. (2003). The Emb proteins of mycobacteria direct arabinosylation of lipoarabinomannan and arabinogalactan via an N-terminal recognition region and a C-terminal synthetic region. Mol. Microbiol..

[39] Al-Jourani O., Benedict S.T., Ross J., Layton A.J., van der Peet P., Marando V.M., Bailey N.P., Heunis T., Manion J., Mensitieri F. (2023). Identification of D-arabinan-degrading enzymes in mycobacteria. Nat. Commun..

[40] Amin A.G., De P., Spencer J.S., Brennan P.J., Daum J., Andre B.G., Joe M., Bai Y., Laurentius L., Porter M.D. (2018). Detection of lipoarabinomannan in urine and serum of HIV-positive and HIV-negative TB suspects using an improved capture-enzyme linked immuno absorbent assay and gas chromatography/mass spectrometry. Tuberculosis.

[41] De P., Amin A.G., Flores D., Simpson A., Dobos K., Chatterjee D. (2021). Structural implications of lipoarabinomannan glycans from global clinical isolates in diagnosis of Mycobacterium tuberculosis infection. J. Biol. Chem..

[42] Angala S., Li W., Boot C.M., Jackson M., McNeil M.R. (2020). Secondary Extended Mannan Side Chains and Attachment of the Arabinan in Mycobacterial Lipoarabinomannan. Commun. Chem..

[43] Turner J., Torrelles J.B. (2018). Mannose-capped lipoarabinomannan in Mycobacterium tuberculosis pathogenesis. Pathog. Dis..

[44] Nigou J., Gilleron M., Puzo G. (2003). Lipoarabinomannans: From structure to biosynthesis. Biochimie.

[45] Palcekova Z., Angala S.K., Belardinelli J.M., Eskandarian H.A., Joe M., Brunton R., Rithner C., Jones V., Nigou J., Lowary T.L. (2019). Disruption of the SucT acyltransferase in Mycobacterium smegmatis abrogates succinylation of cell envelope polysaccharides. J. Biol. Chem..

[46] Chatterjee D., Lowell K., Rivoire B., McNeil M.R., Brennan P.J. (1992). Lipoarabinomannan of Mycobacterium tuberculosis. Capping with mannosyl residues in some strains. J. Biol. Chem..

[47] Chatterjee D., Khoo K.H., McNeil M.R., Dell A., Morris H.R., Brennan P.J. (1993). Structural definition of the non-reducing termini of mannose-capped LAM from Mycobacterium tuberculosis through selective enzymatic degradation and fast atom bombardment-mass spectrometry. Glycobiology.

[48] Khoo K.-H., Tang J.-B., Chatterjee D. (2001). Variation in mannose-capped terminal arabinan motifs of lipoarabinomannans from clinical isolates of *Mycobacterium tuberculosis* and *Mycobacterium avium* complex. J. Biol. Chem..

[49] Angala S.K., McNeil M.R., Shi L., Joe M., Pham H., Zuberogoitia S., Nigou J., Boot C.M., Lowary T.L., Gilleron M. (2017). Biosynthesis of the Methylthioxylose Capping Motif of Lipoarabinomannan in Mycobacterium tuberculosis. ACS Chem. Biol..

[50] Angala S.K., Palcekova Z., Belardinelli J.M., Jackson M. (2018). Covalent modifications of polysaccharides in mycobacteria. Nat. Chem. Biol..

[51] Palcekova Z., Obregon-Henao A., De K., Walz A., Lam H., Philp J., Angala S.K., Patterson J., Pearce C., Zuberogoitia S. (2023). Role of succinyl substituents in the mannose-capping of lipoarabinomannan and control of inflammation in Mycobacterium tuberculosis infection. PLoS Pathog..

[52] Venisse A., Berjeaud J.M., Chaurand P., Gilleron M., Puzo G. (1993). Structural features of lipoarabinomannan from Mycobacterium bovis BCG. Determination of molecular mass by laser desorption mass spectrometry. J. Biol. Chem..

[53] Sada E., Brennan P.J., Herrera T., Torres M. (1990). Evaluation of lipoarabinomannan for the serological diagnosis of tuberculosis. J. Clin. Microbiol..

[54] Cho S.N., Shin J.S., Kim J.D., Chong Y. (1990). Production of monoclonal antibodies to lipoarabinomannan-B and use in the detection of mycobacterial antigens in sputum. Yonsei Med. J..

[55] Sibley L.D., Adams L.B., Krahenbuhl J.L. (1990). Inhibition of interferon-gamma-mediated activation in mouse macrophages treated with lipoarabinomannan. Clin. Exp. Immunol..

[56] Sibley L.D., Hunter S.W., Brennan P.J., Krahenbuhl J.L. (1988). Mycobacterial lipoarabinomannan inhibits gamma interferon-mediated activation of macrophages. Infect. Immun..

[57] Kaplan G., Gandhi R.R., Weinstein D.E., Levis W.R., Patarroyo M.E., Brennan P.J., Cohn Z.A. (1987). Mycobacterium leprae antigen-induced suppression of T cell proliferation in vitro. J. Immunol..

[58] Chan J., Fan X., Hunter S.W., Brennan P.J., Bloom B.R. (1991). Lipoarabinomannan, a possible virulence factor involved in persistence of *Mycobacterium tuberculosis* within macrophages. Infect. Immun..

[59] Moreno C., Mehlert A., Lamb J. (1988). The inhibitory effects of mycobacterial lipoarabinomannan and polysaccharides upon polyclonal and monoclonal human T cell proliferation. Clin. Exp. Immunol..

[60] Moreno C., Taverne J., Mehlert A., Bate C.A.W., Brealey R.J., Meager A., Rook G.A.W., Playfair J.H.L. (1989). Lipoarabinomannan from *Mycobacterium tuberculosis* induces the production of tumor necrosis factor from human and murine macrophages. Clin. Exp. Immunol..

[61] Barnes P.F., Chatterjee D., Brennan P.J., Rea T.H., Modlin R.L. (1992). Tumor necrosis factor production in patients with leprosy. Infect. Immun..

[62] Chatterjee D., Roberts A.D., Lowell K., Brennan P.J., Orme I.M. (1992). Structural basis of capacity of lipoarabinomannan to induce secretion of tumor necrosis factor. Infect. Immun..

[63] Adams L.B., Fukutomi Y., Krahenbuhl J.L. (1993). Regulation of murine macrophage effector functions by lipoarabinomannan from mycobacterial strains with different degrees of virulence. Infect. Immun..

[64] Barnes P.F., Chatterjee D., Abrams J.S., Lu S., Wang E., Yamamura M., Brennan P.J., Modlin R.L. (1992). Cytokine production induced by *Mycobacterium tuberculosis* lipoarabinomannan: Relationship to chemical structure. J. Immunol..

[65] Zhang Y., Rom W.N. (1993). Regulation of the interleukin-1b (IL-1b) gene by mycobacterial components and lipopolysaccharide is mediated by two nuclear factor-IL6 motifs. Mol. Cell Biol..

[66] Maeda N., Nigou J., Herrmann J.L., Jackson M., Amara A., Lagrange P.H., Puzo G., Gicquel B., Neyrolles O. (2003). The cell surface receptor DC-SIGN discriminates between Mycobacterium species through selective recognition of the mannose caps on lipoarabinomannan. J. Biol. Chem..

[67] Schlesinger L.S., Hull S.R., Kaufman T.M. (1994). Binding of the Terminal Mannosyl Units of Lipoarabinomannan from a Virulent Strain of *Mycobacterium Tuberculosis* to Human Macrophages. J. Immunol..

[68] Venisse A., Fournié J.-J., Puzo G. (1995). Mannosylated lipoarabinomannan interacts with phagocytes. Eur. J. Biochem..

[69] Fratti R.A., Chua J., Vergne I., Deretic V. (2003). Mycobacterium tuberculosis glycosylated phosphatidylinositol causes phagosome maturation arrest. Proc. Natl. Acad. Sci. USA.

[70] Schlesinger L.S., Kaufman T.M., Iyer S., Hull S.R., Marchiando L.K. (1996). Differences in mannose receptor-mediated uptake of lipoarabinomannan from virulent and attenuated strains of *Mycobacterium tuberculosis* by human macrophages. J. Immunol..

[71] Sieling P.A., Chatterjee D., Porcelli S.A., Prigozy T.I., Mazzaccaro R.J., Soriano T., Bloom B.R., Brenner M.B., Kronenberg M., Brennan P.J. (1995). CD1-restricted T cell recognition of microbial lipoglycan antigens. Science.

[72] Prigozy T.I., Sieling P.A., Clemens D., Stewart P.L., Behar S.M., Porcelli S.A., Brenner M.B., Modlin R.L., Kronenberg M. (1997). The mannose receptor delivers lipoglycan antigens to endosomes for presentation to T cells by CD1b molecules. Immunity.

[73] Kallert S., Zenk S.F., Walther P., Grieshober M., Weil T., Stenger S. (2015). Liposomal delivery of lipoarabinomannan triggers Mycobacterium tuberculosis specific T-cells. Tuberculosis.

[74] Kaur D., Obregon-Henao A., Pham H., Chatterjee D., Brennan P.J., Jackson M. (2008). Lipoarabinomannan of Mycobacterium: Mannose capping by a multifunctional terminal mannosyltransferase. Proc. Natl. Acad. Sci. USA.

[75] Alderwick L.J., Birch H.L., Mishra A.K., Eggeling L., Besra G.S. (2007). Structure, function and biosynthesis of the Mycobacterium tuberculosis cell wall: Arabinogalactan and lipoarabinomannan assembly with a view to discovering new drug targets. Biochem. Soc. Trans..

[76] Chatterjee D., Brennan P.J., Holst O., Brennan P.J., Itzstein V.M. (2009). Glycosylated components of the mycobacterial cell wall;structure and function. Microbial Glycobiology: Structures, Relevance and Applications.

[77] Briken V., Porcelli S.A., Besra G.S., Kremer L. (2004). Mycobacterial lipoarabinomannan and related lipoglycans: From biogenesis to modulation of the immune response. Mol. Microbiol..

[78] Rivoire B., Ranchoff B., Chatterjee D., Gaylord H., Tsang A., Kolk A.H.J., Aspinall G.O., Brennan P.J. (1989). Generation of monoclonal antibodies to the specific sugar epitopes of *Mycobacterium avium* complex serovars. Infect. Immun..

[79] Ahmad R., Xie L., Pyle M., Suarez M.F., Broger T., Steinberg D., Ame S.M., Lucero M.G., Szucs M.J., MacMullan M. (2019). A rapid triage test for active pulmonary tuberculosis in adult patients with persistent cough. Sci. Transl. Med..

[80] Choudhary A., Patel D., Honnen W., Lai Z., Prattipati R.S., Zheng R.B., Hsueh Y.C., Gennaro M.L., Lardizabal A., Restrepo B.I. (2018). Characterization of the Antigenic Heterogeneity of Lipoarabinomannan, the Major Surface Glycolipid of *Mycobacterium tuberculosis*, and Complexity of Antibody Specificities toward this Antigen. J. Immunol..

[81] Corrigan D.T., Ishida E., Chatterjee D., Lowary T.L., Achkar J.M. (2023). Monoclonal antibodies to lipoarabinomannan/arabinomannan—Characteristics and implications for tuberculosis research and diagnostics. Trends Microbiol..

[82] Lawn S.D., Gupta-Wright A. (2016). Detection of lipoarabinomannan (LAM) in urine is indicative of disseminated TB with renal involvement in patients living with HIV and advanced immunodeficiency: Evidence and implications. Trans. R. Soc. Trop. Med. Hyg..

[83] Gupta-Wright A., Peters J.A., Flach C., Lawn S.D. (2016). Detection of lipoarabinomannan (LAM) in urine is an independent predictor of mortality risk in patients receiving treatment for HIV-associated tuberculosis in sub-Saharan Africa: A systematic review and meta-analysis. BMC Med..

[84] Shah M., Hanrahan C., Wang Z.Y., Dendukuri N., Lawn S.D., Denkinger C.M., Steingart K.R. (2016). Lateral flow urine lipoarabinomannan assay for detecting active tuberculosis in HIV-positive adults. Cochrane Database Syst. Rev..

[85] Amin A.G., De P., Graham B., Calderon R.I., Franke M.F., Chatterjee D. (2021). Urine lipoarabinomannan in HIV uninfected, smear negative, symptomatic TB patients: Effective sample pretreatment for a sensitive immunoassay and mass spectrometry. Sci. Rep..

[86] Amin A.G., De P., Graham B., Jensen B.L., Moreau E., Chatterjee D. (2022). Overcome low levels of detection limit and choice of antibody affects detection of lipoarabinomannan in pediatric tuberculosis. PLoS ONE.

[87] Sigal G.B., Pinter A., Lowary T.L., Kawasaki M., Li A., Mathew A., Tsionsky M., Zheng R.B., Plisova T., Shen K. (2018). A Novel Sensitive Immunoassay Targeting the 5-Methylthio-d-Xylofuranose-Lipoarabinomannan Epitope Meets the WHO’s Performance Target for Tuberculosis Diagnosis. J. Clin. Microbiol..

[88] Zhang A., Jumbe E., Krysiak R., Sidiki S., Kelley H.V., Chemey E.K., Kamba C., Mwapasa V., Garcia J.I., Norris A. (2018). Low-cost diagnostic test for susceptible and drug-resistant tuberculosis in rural Malawi. Afr. J. Lab. Med..

[89] Lawn S.D., Kerkhoff A.D., Nicol M.P., Meintjes G. (2015). Underestimation of the true specificity of the urine lipoarabinomannan (LAM) point-of-care diagnostic assay for HIV-associated tuberculosis. J. Acquir. Immune Defic. Syndr..

[90] Paris L., Magni R., Zaidi F., Araujo R., Saini N., Harpole M., Coronel J., Kirwan D.E., Steinberg H., Gilman R.H. (2017). Urine lipoarabinomannan glycan in HIV-negative patients with pulmonary tuberculosis correlates with disease severity. Sci. Transl. Med..

[91] Nabeemeeah F., Sabet R., Moloantoa T., Waja Z., Pretorius Z., Majoro K., Letutu-Xaba M., Vilaplana C., Nigou J., Martinson N. (2023). Exhaled breath specimens subjected to point-of-care lipoarabinomannan testing. Int. J. Tuberc. Lung Dis..

[92] Mosquera-Restrepo S.F., Zuberogoïtia S., Gouxette L., Layre E., Gilleron M., Stella A., Rengel D., Burlet-Schiltz O., Caro A.C., Garcia L. (2022). A Mycobacterium tuberculosis fingerprint in human breath allows tuberculosis detection. Nat. Commun..

[93] Broger T., Tsionksy M., Mathew A., Lowary T.L., Pinter A., Plisova T., Bartlett D., Barbero S., Denkinger C.M., Moreau E. (2019). Sensitive electrochemiluminescence (ECL) immunoassays for detecting lipoarabinomannan (LAM) and ESAT-6 in urine and serum from tuberculosis patients. PLoS ONE.

[94] De P., Amin A.G., Graham B., Martiniano S.L., Caceres S.M., Poch K.R., Jones M.C., Saavedra M.T., Malcolm K.C., Nick J.A. (2020). Urine lipoarabinomannan as a marker for low-risk of NTM infection in the CF airway. J. Cyst. Fibros..

[95] Qvist T., Johansen I.S., Pressler T., Hoiby N., Andersen A.B., Katzenstein T.L., Bjerrum S. (2014). Urine lipoarabinomannan point-of-care testing in patients affected by pulmonary nontuberculous mycobacteria--experiences from the Danish Cystic Fibrosis cohort study. BMC Infect. Dis..

[96] Nick J.A., Dedrick R.M., Gray A.L., Vladar E.K., Smith B.E., Freeman K.G., Malcolm K.C., Epperson L.E., Hasan N.A., Hendrix J. (2022). Host and pathogen response to bacteriophage engineered against Mycobacterium abscessus lung infection. Cell.

[97] Guerardel Y., Maes E., Briken V., Chirat F., Leroy Y., Locht C., Strecker G., Kremer L. (2003). Lipomannan and lipoarabinomannan from a clinical isolate of *Mycobacterium kansasii*: Novel structural features and apoptosis-inducing properties. J. Biol. Chem..

[98] Glass L.N., Swapna G., Chavadi S.S., Tufariello J.M., Mi K., Drumm J.E., Lam T.T., Zhu G., Zhan C., Vilcheze C. (2017). Mycobacterium tuberculosis universal stress protein Rv2623 interacts with the putative ATP binding cassette (ABC) transporter Rv1747 to regulate mycobacterial growth. PLoS Pathog..

[99] Coscolla M., Gagneux S. (2014). Consequences of genomic diversity in Mycobacterium tuberculosis. Semin. Immunol..

[100] Gagneux S., DeRiemer K., Van T., Kato-Maeda M., de Jong B.C., Narayanan S., Nicol M., Niemann S., Kremer K., Gutierrez M.C. (2006). Variable host-pathogen compatibility in *Mycobacterium tuberculosis*. Proc. Natl. Acad. Sci. USA.

[101] Mikusova K., Huang H., Yagi T., Holsters M., Vereecke D., D’Haeze W., Scherman M.S., Brennan P.J., McNeil M.R., Crick D.C. (2005). Decaprenylphosphoryl arabinofuranose, the donor of the D-arabinofuranosyl residues of mycobacterial arabinan, is formed via a two-step epimerization of decaprenylphosphoryl ribose. J. Bacteriol..

[102] Brock M., Hanlon D., Zhao M., Pollock N.R. (2020). Detection of mycobacterial lipoarabinomannan in serum for diagnosis of active tuberculosis. Diagn. Microbiol. Infect. Dis..

[103] Arcos J., Sasindran S.J., Fujiwara N., Turner J., Schlesinger L.S., Torrelles J.B. (2011). Human lung hydrolases delineate Mycobacterium tuberculosis-macrophage interactions and the capacity to control infection. J. Immunol..

